# “… Exercise opportunities became very important”: Scottish older adults’ changes in physical activity during Covid19’

**DOI:** 10.1186/s11556-022-00295-z

**Published:** 2022-07-02

**Authors:** Simone A. Tomaz, Gemma C. Ryde, Bridgitte Swales, Kacey C. Neely, Federico Andreis, Pete Coffee, Jenni Connelly, Andrew Kirkland, Louise McCabe, Karen Watchman, Jack G. Martin, Ilaria Pina, Anna C. Whittaker

**Affiliations:** 1 Faculty of Health Sciences and Sport, University of Stirling, Stirling, FK9 4LA UK; 2 Nesta, The Bayes Centre 47, Potterrow, Edinburgh, EH8 9BT UK

**Keywords:** Exercise, Walking, Sedentary, Copula model, GAM, Qualitative

## Abstract

**Background:**

The purpose of this study was to explore perceived changes in physical activity (PA) due to Covid19 stay-at-home and social distancing guidance among older adults.

**Methods:**

Participants (*n* = 1429, 77% female, 84% ≥60 years) living in Scotland completed an online survey in Summer 2020 measuring PA and wellbeing (indexed through loneliness, and health-related quality of life). The survey included open- and closed-ended questions about how these variables changed in response to Covid19 social distancing and ‘shielding’ guidelines.

**Results:**

From the International Physical Activity Questionnaire (IPAQ), the majority reported high volumes of PA, indicative of being ‘moderately’ or ‘highly’ active. When asked specifically about strength training, 12% reported engagement on ≥2d/wk. Most participants reported that PA had changed during this time, citing reduced use of exercise facilities, increased active travel, and online PA classes; although only 16% reported engaging in PA online.

**Conclusions:**

Higher levels of PA were found to be associated with better health-related quality of life. Additional efforts should be made to support PA engagement in older adults, including strength training and other tailored approaches to support individual needs.

**Supplementary Information:**

The online version contains supplementary material available at 10.1186/s11556-022-00295-z.

## Introduction

The benefits of physical activity are well established for all ages, and older adults benefit from physical activity in a variety of ways. Older adults who are physically active have been shown to have improved heart health [1], cognitive function [2], stronger bones [3], and better mental health [4]. Physical activity can counteract the negative health effects associated with ageing [5]. For example, it can delay the onset of dementia [6], help prevent falls [7, 8] and allow older adults to maintain independence and activities of daily living [9, 10]. Review evidence also suggests that social support among older adults can be positively influenced by physical activity [11], with the World Health Organisation (WHO) identifying social support as a key determinant of active ageing [12].

Coronavirus or severe acute respiratory syndrome coronavirus 2 (SARS-CoV-2), that causes the coronavirus disease (Covid19), was declared a pandemic by the World Health Organization on 11th March 2020 [13]. In the UK, the number of confirmed cases on the 5th of May 2020 was 195,435, the highest in Europe and 2nd highest globally. On this day, 30,929 cumulative Covid19 related deaths had been recorded [14]. Older adults in the UK have arguably been among the hardest-hit groups in terms of confirmed cases and deaths. In addition to being one of the hardest-hit nations, adults in the UK were given differing levels of guidance according to their risk. Initial guidance that came into force in March 2020 for the UK included: “*The British public are instructed that they must stay at home, except for certain “very limited purposes” – shopping for basic necessities; for “one form of exercise a day”; for any medical need; and to travel to and from work when “absolutely necessary””* [15]. This period was referred to as ‘lockdown’*.* It was encouraging that physical activity was a priority on the agenda and frequently communicated in the daily UK government bulletins. However, persons who were ‘shielding’ were advised to not leave their homes and to also ‘socially distance’ themselves from individuals within their home. This meant that for at least 12 weeks, adults over 70 years and persons with pre-existing conditions were advised to stay at home for their protection.

Multiple studies from other countries have reported findings that suggest older people experienced changes in physical activity as a result of the pandemic or the restrictions imposed by government, including Finland [16], France [17], the Netherlands [18] and Canada [19]. One study focused on adults (18 years and older) in Scotland reported on changes in physical activity, sleep and sedentary behaviour. This study demonstrated that physical activity levels (walking, MVPA) did change as restrictions in Scotland altered [20]. As such, physical activity has been identified as a key contributor to the health of older people in the context of the Covid19 pandemic [21]. However, it is important to note that even prior to the pandemic, older adults with common morbidities associated with ageing, such as frailty and hearing or sight impairment [22] were likely to experience difficulties in being physically active, making them particularly vulnerable to the impact of low(er) levels of physical activity during the pandemic. This is particularly true for older people in Scotland. Findings from the Scottish Health Survey [23] suggest that 34% of all adults were not engaged in enough MVPA to positively influence their health. The proportion of older adults meeting the MVPA component of physical activity guidelines was 53% for those aged 65–74 years, and only 31% for those aged 75 years and over [23]. Lastly, amongst adults taking part in any physical activity, only 8% of those aged 75 and over meet both the MVPA and muscle strengthening recommendations [23]; this was the lowest out of any age group. These levels are concerning and are of even greater concern in the context of stay-at-home orders and social distancing restrictions due to the global Covid19 pandemic. Even older adults without pre-existing conditions who were not advised to shield would have still had limited opportunities to be physically active due to the shutting down of leisure and recreational facilities [24].

Consequently, the aim of the present study was to examine the perceived impact of social distancing during the Covid19 pandemic on physical activity and wellbeing, specifically loneliness and health-related quality of life, in older adults. In the context of this paper, we refer to ‘social distancing’ while acknowledging that it is/was not only the guidance to stay 2 m away from another person that necessarily restricts engagement in physical activity, but also the restrictions placed specifically on individuals advised to shield (in the form of stay-at-home orders), as well as the closure of leisure facilities (such as gyms and public swimming pools) as social distancing would not be possible. Specifically, the objectives were to: 1) examine self-reported physical activity during social distancing between May and July 2020; 2) determine perceived changes in physical activity from before the pandemic to during social distancing and other restrictions (using both quantitative and qualitative methods); 3) explore associations between physical activity and health-related quality of life and loneliness during social distancing; 4) explore the reasons for changes in physical activity and identify strategies used to remain physically active.

## Methods

### Participants and design

**Fig. 1:**
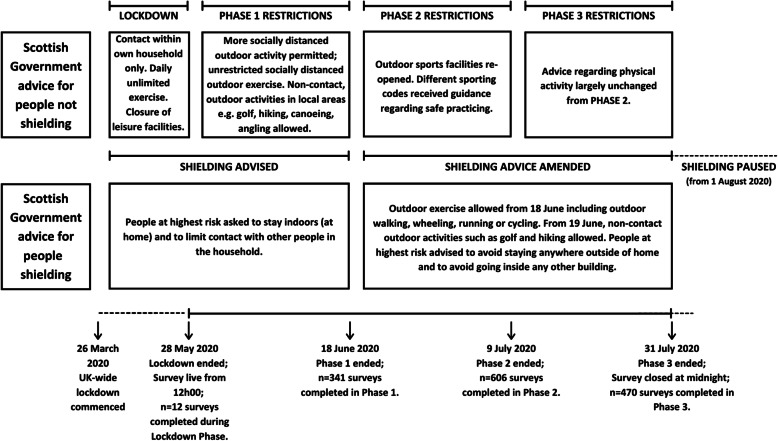
Scotland lockdown phases in summer 2020 in relation to data collection for this study. Advice comparison for persons not shielding (top) and persons advised to shield (bottom)

This project used a concurrent mixed methods survey (where the questionnaire included both open- and closed-ended questions) to explore the perceived impact of social distancing on physical activity, loneliness, health-related quality of life and social activity including social support. This paper focusses on and describes the findings pertaining to physical activity. The detailed findings regarding loneliness, social activity and social support have been presented elsewhere, including recruitment details [25]. Where pertinent to this analysis, methods are detailed in this manuscript. The online survey was hosted in JISC software and targeted adults aged 60 years and over, but also included other vulnerable or ‘at risk’ groups to such as those who were carers or had a learning disability or were shielding. The survey went live on the morning of the 28th of May and was distributed across Scotland via social media, emails, and snowballing through personal contacts. Later this same day, the Scottish First Minister had announced the move from ‘Lockdown’ to ‘Phase 1’. From the 27th of June, targeted Facebook advertising was used centring around major cities in Scotland, focusing on those aged from 45 years to capture individuals who might see it and be able to pass it on to an older relative or friend. The survey closed on the 31st of July, also the day after the Scottish Government announced that ‘shielding’ would be paused from 1st August. The detail of the phases and the relevance to physical activity is shown in Fig. 1. Sampling was non-probabilistic, and although the survey was completed by 1429 respondents.

### Measures

#### Socio-demographics

Socio-demographic variables of interest included age, sex, ethnicity, relationship status, education, income bracket, employment status, postcode, number of people in the home, and number of people requiring care within the home as well as a question about whether the participant was a care provider. Where a participant responded with ‘Prefer not to say’ for any of these variables, the data were marked as ‘missing’. There were eight categories of income bracket spanning from <£2500 to more than £50 k per year. In order to determine urban and rural living, participants that provided postcodes were classified using the Scottish Government Urban Rural Classifications Breakdown [26]. The 3-fold classification (‘rest of Scotland’ indicating urban and two rural categories: accessible rural and remote rural) is presented and used in the analyses, as previously by others [27].

#### Physical activity, sedentary behaviour, screen time and sleep

Physical activity was assessed using the short International Physical Activity Questionnaire (IPAQ) [28]. This has been shown to be valid among older adults [29] but we also followed guidelines published as part of this validation such as the option for people to complete a breakdown of daily activities to improve recall. The IPAQ data were cleaned according to the IPAQ User Manual [30] which codes vigorous, moderate, and walking physical activity, as well as sitting time. In scoring the IPAQ, there are three levels of physical activity classification: ‘low’, ‘moderate’ or ‘high’. A low active participant has reported physical activity that reflects not meeting physical activity guidelines. Participants in the moderate activity category are those who report a level of physical activity of at least 30 minutes of moderate-intensity physical activity on most days, thus meeting physical activity guidelines. Highly active participants reported at least one hour of moderate-intensity physical activity or half an hour of vigorous-intensity physical activity per day, thus achieving levels of physical activity that well exceed the physical activity guidelines. Participants were also asked whether each of these levels of physical activity and sitting time was less, the same or more than before social distancing.

In addition to these standard IPAQ questions, participants were also asked about light-intensity physical activity (described as *activities that do not cause you to sweat or become short of breath and are often referred to as lifestyle activities. Light activities may include gardening, housework or a casual cycle done solely for recreation, sport, exercise, or leisure where you are moving but are not out of breath. During the last 7 days, on how many days did you engage in light activity? Do not include walking*) and number of days engaged in strength training (described as *strength-based or body weight exercises like training with free weights, a weight machine, or resistance bands so that you can lift more weight, or for the purpose of building muscle*). The data for the light-intensity physical activity were cleaned according to the IPAQ guidelines with the exception that all light physical activity data exceeding 360 min/day (6 hr./day) were truncated to 360 min/day (*n* = 26) and the strength training data were captured only as number of days (in the past seven days). For both light intensity physical activity and strength training, participants were asked if their activity was less, the same, or more, compared to before social distancing began. Additionally, participants who reported two or more days of strength training were also coded as meeting PA guidelines. Screen time was assessed in a similar manner to the IPAQ questions through additional questions regarding the number of days engaged in screen time and average minutes per day. Participants were also asked whether their screen time was less, the same or more than before social distancing.

Participants reported on their typical bedtime, waking up time, hours of sleep (time in bed) and sleep quality. The questions in the survey were asked in a ‘drop down’ menu in 30 min-intervals (bedtime and waking up) and in hour-long intervals (time in bed). Participants were able to answer ‘other’ and manually enter a time if there was not a time applicable to them to report. The answers for the bedtime and waking up questions were converted to decimals so that an approximate average for both could be reported (e.g., 8 pm = 20.0 = 20 h00; 7:30 am = 7.5 = 07 h30). The answers for time in bed were used to place participants into categories according to the National Sleep Foundation sleep time recommendations [31] for those aged under 65 years, and those aged 65+ years, which split participants into five categories: short sleepers; short but may be appropriate sleepers; ideal sleepers; long but may be appropriate sleepers; and long sleepers. These were collapsed into three categories of short (defined as sleeping less than ideal), ideal, and long sleepers (defined as sleeping more than ideal) for the purpose of analysis due to the skewness of the data. Participants were also asked whether since before social distancing, they were sleeping less well, the same, or better. For all physical activity, sitting time, screen time and sleep questions, if participants provided a range of time in a particular activity (e.g., 4–5 hours of screen time per day), the median of the range was determined and used in analysis.

#### Psychosocial variables

Although the focus of this paper is on physical activity, the overall project aimed to explore the effect of social distancing on loneliness, health-related quality of life and social activity including social support. The methods (and results) for loneliness and social activity including social support are described in greater detail elsewhere [25] and are included here as they are relevant for the quantitative data analysis: Loneliness was measured using the revised brief form of the UCLA Loneliness scale (ULS-6) [32]. Health-related quality of life was measured using the EQ. 5D-3L [33] which assesses mobility, self-care, usual activities, pain/discomfort, and anxiety/depression on three-point scales to indicate level of problems (no difficulty, some difficulty, considerable difficulty). It also includes a 0–100 rating of health. Participants were also asked whether their anxiety/depression and their state of health was worse, the same or better than before social distancing. For social activity, participants were asked about the number of days they engaged in social activities (in the last 7 days) and average time spent in social activity (in minutes) using similar wording to that of the IPAQ for consistency of question style and in the absence of a brief measure of this type. Social network size was estimated using a question from the Medical Outcomes Survey Social Support Scale [34] which asks about number of close friends and relatives that one can feel at ease with and talk about what is on their mind. Perceived social support was measured using a brief perceived social support questionnaire (BPSSQ) [35].

#### Changes in physical activity and strategies to engage in physical activity (qualitative)

The survey included five open-ended questions, one of which is not addressed in this manuscript as it was targeted to specific changes in social activity (reported elsewhere [25]:). The first 3 questions were asked following the IPAQ and additional physical activity questions: *1) Have you started to do any new physical activities since social distancing started, such as online exercise classes, walking round the garden or walking to places instead of using a car or bus? If yes, please tell us about what you have been doing. You may be as descriptive as you want.; 2) Have you changed the way you do physical activity, such as attending exercise classes online instead of in person, walking or cycling to place more often than before or completely changed your routine? If yes, please tell us about the changes you have made. You may be as descriptive as you want; and 3) Where do you currently do your physical activity? (please choose all which apply; options: house, online, garden, local area, other); If you selected Other, please specify.* The last question of the survey was also considered for analysis: *4) Is there anything else that you can think of about social engagement, loneliness, wellbeing and/or physical activity during the pandemic that we haven’t covered in this survey? You may be as descriptive as you want.* For this manuscript, only responses that overtly mention physical activity are reported.

#### Ethical approval

Ethical approval for this study was obtained from the General University Ethics Panel (GUEP 905) and informed consent was given on the first page of the online survey or orally for the telephone version. All participants provided consent for their participation by agreeing to complete the survey. This study adheres to the guidelines described in the Declaration of Helsinki Ethical Principles for Medical Research Involving Human Subjects [36].

### Data analysis

#### Quantitative component

The dataset was pre-processed in Microsoft Excel, SPSS v26 and R 4.0.2 (R Core Team, 2020). Analyses were carried out using R 4.0.2 [37]. Descriptive statistics were obtained for all variables and all individuals, whereas the non-probabilistic nature of the design made it possible to attempt inference only for the 60+ years group, for which there was enough background information with respect to the target population to assess representativeness. A bivariate Clayton copula model [38] with Gamma margins described by Generalised Additive Models [39] was adopted to explore associations between variables (including physical activity) with health-related quality of life (EQ. 5D-3L) and loneliness (UCLA) jointly. This analysis relies on the same modelling approach adopted elsewhere [25], while at the same time aiming at investigating more in detail aspects related to physical activity. The two outcomes (loneliness and health-related quality of life) were observed to be moderately associated, with an estimated Kendall’s tau of ~ 0.22. Covariates considered for both outcomes included age (years), deprivation (using SIMD quintiles), rurality (3-fold definition), sex (binary), social support (BPSSQ), size of social network, social time (hours per week), perceived health rating, screen time (hours per day), reported walking (minutes per week for the past week), and sleep category (3-fold). These variables were selected based on existing literature (age, deprivation, sex, social support, physical activity, and sleep), and have previously been shown to be associated with physical activity and/or wellbeing (loneliness and health-related quality of life). Potentially nonlinear relationships (concerning numerical and ordinal variables) were catered for by using splines, whereas categorical covariates entered the model as linear terms.

#### Qualitative component

Qualitative data were exported from the Online Surveys (formerly BOS) software into Excel. Thereafter, data were imported from Excel to NVIVO v12 Pro for Windows. ST coded and KN and BS acted as ‘critical buddies’ during the coding process, using thematic analysis that involved both inductive and deductive approaches [40]. For questions 1 and 2, the specific nature of the changes in physical activity and how and why these changes occurred were explored. For these questions, an explanatory approach to the qualitative analysis of the answers was adopted, such that the qualitative data were used to explain or substantiate quantitative findings. Another reason for this approach included the tendency for participants to answer these two open-ended questions with responses that did not provide much insight into the differences or changes in physical activity (e.g., participants would answer with one or two words such as “Walking”, “Online”, “Gardening”), or participants would provide overlapping or sometimes identical answers to questions 1 and 2. Answers were grouped according to the following themes, which may be interpreted as explanations or reasons for new and/or changed physical activity, reported in descending order: 1) active travel, 2) access to facilities, 3) optimised walking, 4) health and injury, 5) shielding, 6) pets, 7) weather, 8) caring, 9) time availability, and 10) employment. Additionally, this study aimed to investigate any answers that reflected successful strategies to maintain physical activity, including positive changes made because of lockdown, as well as the role of setting goals or targets. For question 3, the responses regarding where physical activity took place were first considered according to the four options provided (i.e., house, online, garden, local area). Thereafter, the responses to the ‘other’ section were considered as qualitative data because it was evident that many of the responses of those who selected ‘other’ had provided additional detail regarding their location and the reasons for their choice, beyond simply providing an additional location. For question 4, the answers were primarily scanned for details pertaining to earlier questions in the survey and where appropriate, those answers were addressed (e.g., postcode difficulties, missing options for some questions). Thereafter, the answers were read by ST and coded according to common themes that provide insight to the lived experiences of all participants. For this paper, only responses regarding physical activity are included as other themes (e.g., grief and sadness, anxiety, feelings of frustration) are beyond the scope of this manuscript and reported elsewhere [25]. To remain consistent with quantitative analysis, only qualitative data for participants aged 60 and over are presented.

## Results

### Data missingness and representativeness

The survey was completed by 1429 respondents residing in Scotland. Missingness across the dataset was low at 3.9% of the total observations spread across approximately 53% of variables; see [25] for more details. Participants were slightly imbalanced with respect to deprivation using the SIMD quintiles; the most deprived group is proportionally under-represented with respect to the reference population (persons 60 years and over in Scotland), and the least deprived quintile appears to be over-represented, whereas we observe an over-representation of the least deprived quintile. The second to fourth quintiles appear to be fairly represented. This imbalance is likely due to both the surveying method (using predominantly online recruitment methods) and the differing non-response rates by socio-economic status as is commonly reported [41]. Therefore, the imbalance needs to be considered when interpreting the results.

For those under the age of 60 years, the reference population (defined as individuals self-identifying as ‘at-risk’ or vulnerable to Covid-19) is such that very little, if any, information exists about the key variables, thus making direct comparisons to assess sample balance across them unreliable or impossible. Therefore, although data were collected on participants representing a wider age range (22–98 years), only data on participants aged 60+ are presented in this paper; these were 84% (*n* = 1198) of the original 1429 participants that completed the survey.

### Descriptive statistics

#### Socio-demographics and health behaviours

Participant characteristics and socio-demographics are shown in Table 1. The mean (SD) age was 67.3 (5.4) years; participants were aged between 60 and 98 years although the majority were between 60 and 70 years; median and IQR 66 (63–71). The majority reported their ethnicity as ‘White British’ (*n* = 1185, 99%) and reflects the Scottish population [42]. Most participants also fitted into the ‘retired’ (*n* = 811, 68%) or ‘Employed/self-employed’ (*n* = 214, 18%) categories, and two income brackets earning <£10,000 *n* = 106, 11%; earning ≥ £30,000 *n* = 376, 38%; ‘Prefer not to say’ *n* = 194). Of the 52% with a diagnosis of a health condition, 94% were taking prescribed medication for it. Most participants reported being non-smokers (95%).

**Table 1:** Sociodemographic characteristics of the sample aged > 60 years (*n* = 1198)

Variable		Total n	n (%)
**Gender**	Female^a^	1196	920 (77)
**Relationship**	Single	1191	78 (7)
	Divorced/widowed		306 (26)
	In a relationship		53 (4)
	Married/cohabiting		754 (63)
**Health condition**	Yes	1198	624 (52)
	Two or more		170 (14)
**Education**	Did not complete	1392	72 (6)
	GCSE/O-levels^b^		113 (10)
	Post-16 vocational course		40 (3)
	Highers/A-levels^b^		121 (10)
	Undergraduate degree^b^		545 (47)
	Postgraduate degree		277 (24)
**SIMD deprivation quintile**^c^	1 (most deprived)	1094	89 (8)
	2		142 (13)
	3		229 (21)
	4		282 (26)
	5 (least deprived)		351 (32)
**Urban/rural 3-fold classification**^d^	‘Rest of Scotland’	1094	826 (69)
	Accessible rural		199 (17)
	Remote rural		69 (6)

^1^Two participants selected ‘prefer not to say’ in response to the gender question

^2^Or equivalent

^3^SIMD values determined using valid post codes (*n* = 104, 9% did not provide a valid Scotland postcode)

^4^Rest of Scotland’ includes Large Urban Areas, Other Urban Areas, Accessible Small Towns, and Remote Small Towns

#### Physical activity, sedentary behaviour, screen time and sleep

Physical activity, sedentary behaviour, screen time and sleep data are displayed in Table 2. In accordance with the IPAQ manual, total physical activity derived from the IPAQ variables (including vigorous, moderate, and walking) was used to determine physical activity categories for each participant shown in Table 3. As such, three-quarters of the participants reported physical activity volumes that categorized them as meeting physical activity guidelines. With regards to strength training, the overwhelming majority (87%) reported engaging in strength training less than two days weekly and therefore did not meet the strength training component of the physical activity guidelines. The participants’ reported time in bed was used to determine sleep categories for each participant based on the National Sleep Foundation recommendations. A minority of the participants were classified as ‘long sleepers’, with a relatively even split of participants across the sample being categorised as either ‘ideal sleepers’ or ‘short sleepers’.

**Table 2:** Physical activity, sedentary behaviour, screen time and sleep descriptive results, overall and stratified by gender

	n	All	Men	Women
**Weekly PA from IPAQ:**				
Vigorous PA (min/wk)	1193	30.0 (0, 180.0)	45.0 (0, 210.0)	20.0 (0, 140.0)
Moderate PA (min/wk)	1193	90.0 (0, 240.0)	120.0 (0, 300.0)	80.0 (0, 240.0)
Walking PA (min/wk)	1193	270.0 (100.0, 420.0)	270.0 (120.0, 450.0)	240.0 (80.0, 420.0)
Total PA (min/wk)	1193	525.0 (240.0, 900.0)	590.0 (290.0, 1020.0)	477.5 (180.0, 840.0)
**Other PA and SB variables:**				
Light PA: (min/wk)	1195	307.5 (140.0, 630.0)	210.0 (80.0, 420.0)	300.0 (120.0, 630.0)
Screen time hours per day	1193	3.0 (2.0, 5.0)	3.0 (2.0, 5.0)	3.0 (2.0, 5.0)
Sitting time hours per day	1191	5.0 (3.0, 7.0)	5.0 (3.0, 8.0)	5.0 (3.0, 8.0)
**Sleep variables**				
Bedtime (hh:mm)	1188	23:00 (22:30, 23:30)	23:00 (22:30, 00:00)	23:00 (22:30, 23:30)
Wake up time (hh:mm)	1188	08:00 (07:00, 08:30)	08:00 (07:00, 08:30)	8:00 (07:00, 09:00)

*PA* Physical activity, *IPAQ* International Physical Activity Questionnaire

All variables presented as Median (25th, 75th percentile)

**Table 3:** Proportion of participants meeting physical activity and sleep guidelines, stratified by gender

	All	Men	Women
**IPAQ Physical activity category**^b^			
Low PA	252 (21)	40 (14)	212 (23)
Mod PA	427 (36)	99 (36)	328 (36)
High PA	515 (43)^a^	136 (49)	377 (41)
**Strength training category**			
Yes (≥2d)	155 (13)^a^	38 (14)	115 (12)
No	1044 (87)	238 (86)	806 (88)
**Meeting both components of PA guidelines**			
Yes	140 (12)^a^	36 (13)	102 (11)
No	1054 (88)	239 (87)	815 (89)
**Sleep category**^c^			
Short sleeper	520 (44)^a^	96 (35)	423 (46)
Ideal sleeper	616 (52)^a^	164 (60)	451 (50)
Long sleeper	51 (4)	15 (5)	36 (4)

^a^Two participants selected ‘prefer not to say’ in response to the gender question; frequencies do not add up

^b^Low PA = Not meeting PA guidelines; Mod = meeting PA guidelines; High = meeting PA guidelines but highly active

^c^Short sleepers reported less sleep than ideal, long sleepers reported more sleep than ideal

#### Health-related quality of life, loneliness, and social support/contact

On the EQ. 5D-3L, participants reported moderate health-related quality of life, and self-reported health rating; the total scores for this as well as mean loneliness score, social network size, perceived social support, and social contact time are shown in Table 4. The present sample appeared to report better mobility, self-care, and ability to perform usual activities than normative values for age group, but more problems with pain and anxiety/depression [43]. Table 4 also reports on the variables included in the models. These variables (loneliness, social support and social contact) have been discussed in greater detail elsewhere [25].

**Table 4:** Scores for psychosocial variables included in the model

Variable	Mean (SD)
EQ 5D-3L score (out of 15)^a^	6.7 (1.6)
Current health rating (out of 100)	72.5 (19.9)
Loneliness score (out of 24)	12.7 (4.7)
Perceived Social Support score (average out of 6)	3.8 (1.0)
Social network size	5.5 (5.1)
Social contact (days per week)	5.4 (1.9)
Social time (hours per week)	7.0 (8.7)

^a^A higher score = poorer health

#### Change in physical activity, sedentary behaviour, screen time, and sleep

Perceived changes from before social distancing began are shown for all variables where this question was asked in the 60+ years group in Table 5. Reported changes in vigorous- and moderate-intensity physical activity were similar with approximately half of the participants reporting having similar pre-social distancing levels. Just over a quarter of the participants reported walking more since social distancing. For strength training, 22% of participants reported doing less compared to before social distancing, while the majority of the participants reported that their strength training levels were the same, which for the majority was less than the two days per week guidelines already. For both screen time and sitting time, most participants reported doing more compared to before social distancing.

**Table 5:** Perceived changes since pre-social distancing for participants (*n* = 1198)

Variable	Less	Same	More
IPAQ vigorous PA	462 (39)	553 (46)	183 (15)
IPAQ moderate PA	460 (38)	579 (48)	159 (13)
IPAQ walking time	458 (38)	417 (35)	323 (27)
Light PA	329 (27)	717 (60)	152 (13)
Strength training time	262 (22)	869 (72)	67 (6)
Sitting time	80 (7)	442 (37)	676 (56)
Screen time	42 (4)	416 (35)	740 (62)
Sleep volume	328 (27)	801 (67)	69 (6)

All data presented as n (%)

### Association of physical activity with loneliness and health-related quality of life

#### Categorical predictor variables

**Fig. 2:**
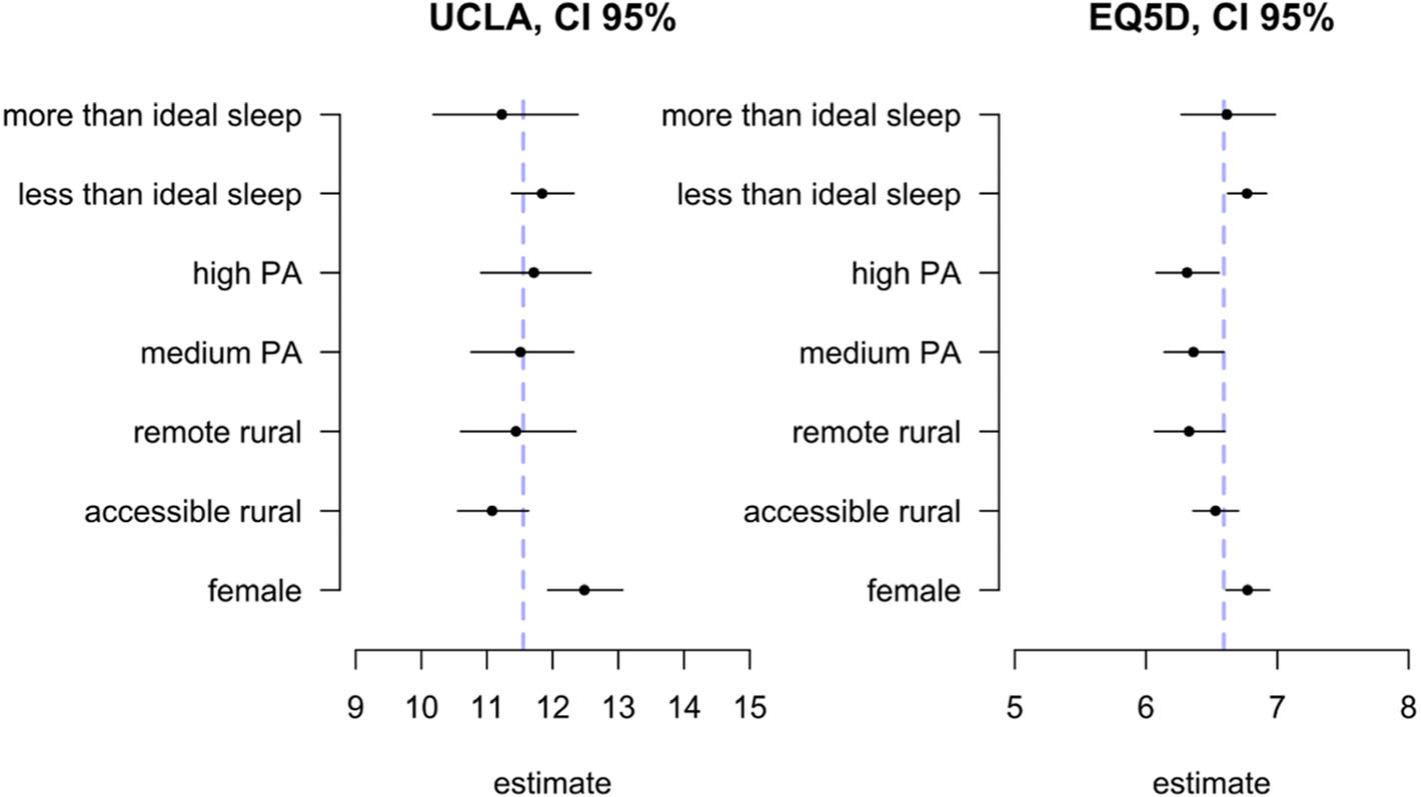
Parameter estimates on the outcome scale for the effect of categorical variables included in the model, by outcome (UCLA Loneliness and EQ. 5D health-related quality of life). The dashed line indicates the model intercept (the model average when everything is kept at the reference category)

In this manuscript, associations with the physical activity variables (physical activity category, reported walking time) is the focus. Figure 2 presents estimate from the joint model looking at associations between categorical predictor variables with loneliness and health-related quality of life scores, on the outcome scale.

Figure 2 shows that being female related to higher loneliness and worse health-related quality of life. Further, being in the high or medium active physical activity category was associated with better health-related quality of life (lower EQ. 5D score). Physical activity level was not associated with loneliness. Finally, living in a remote rural location was associated with better health-related quality of life, but less than ideal sleep was related to worse health-related quality of life.

#### Scale predictor variables

**Fig. 3:**
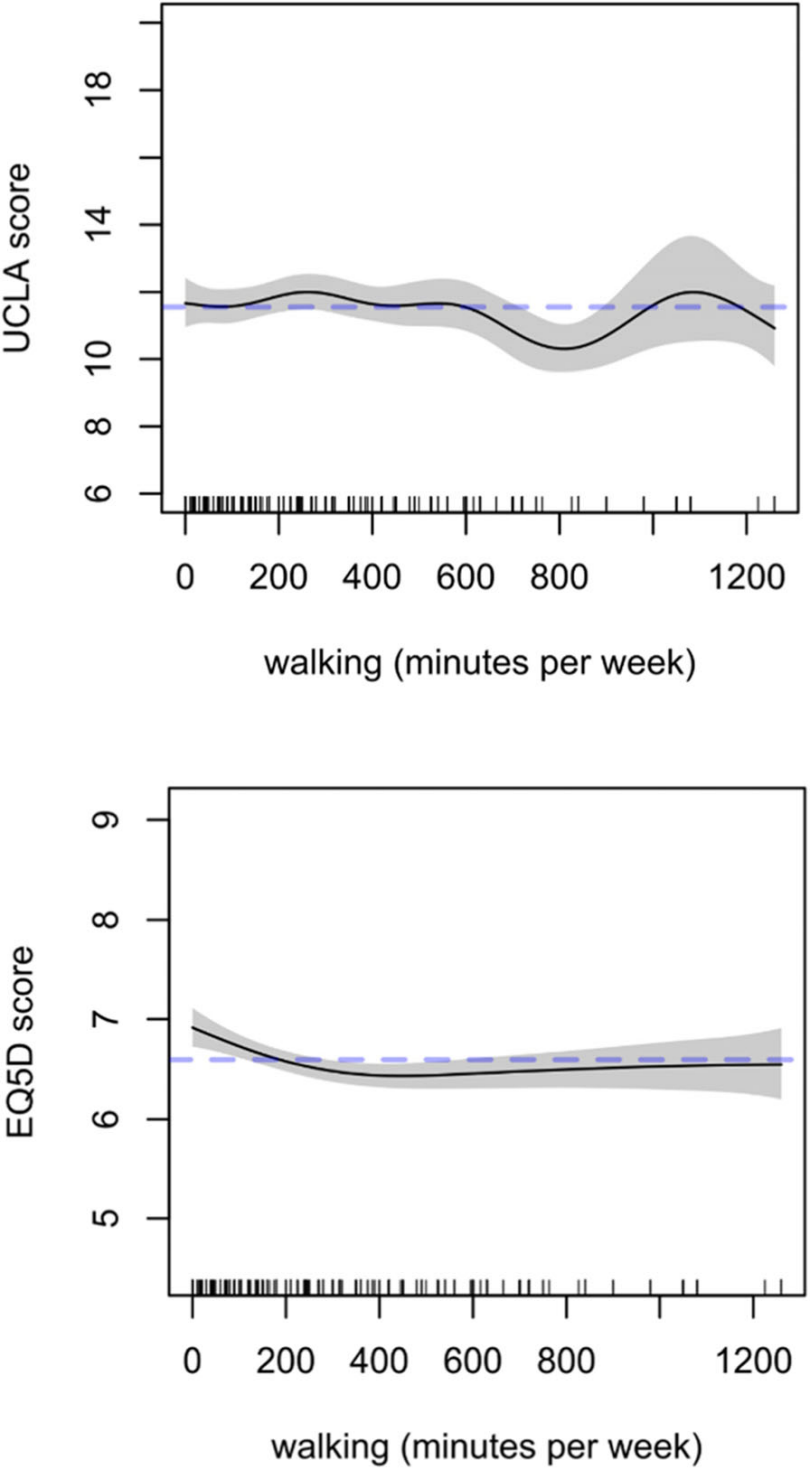
Spline plots showing the estimated relationship between health-related quality of life (EQ. 5D, top) and loneliness (UCLA, bottom) with walking; the dashed line indicates the model average when all other variables are kept at the average or reference level

Figure 3 presents plots of the estimated shape of the relationship (spline plot) between walking (min/wk) with the two outcomes. The number and location of observations across the range of the independent variable is also shown as a density rug; the grey shaded bands indicate uncertainty around the estimates. Supplementary File 1 contains the complete output of the statistical model, while Supplementary File 2 contains all the spline plots based on it.

With reference to Fig. 3, reported walking of less than ±150 minutes per week had no association with loneliness scores. Between roughly 200 and 300 minutes of walking per week appeared to be associated with higher loneliness scores. Walking more than 600 minutes per week was associated with a lower UCLA score, although this is based on a limited number of data points, most of the sample reported walking less than 200 minutes per week. Greater volumes of reported walking time appeared to be associated with a lower EQ. 5D score (indicating better health-related quality of life), although very high volumes of walking (in excess of ±500 minutes per week) did not appear to yield better health-related quality of life scores in comparison to walking ±300 minutes per week.

### Qualitative results

#### Changes in physical activity and strategies to engage in physical activity (qualitative)

Overall, the responses to the open-ended questions in the survey indicated that there was substantial variation in not only the experience of social distancing (reported previously [25]), but also in the changes to physical activity and strategies used to engage in physical activity. These variations appeared to differ on an individual basis and the qualitative data presented indicates the complexity of the effect of social distancing guidelines (in addition to stay-at-home orders and closure of leisure facilities) on adults aged over 60 years.

The most common change in physical activity and/or most commonly reported ‘new’ physical activity was active travel. Many participants reported changing or replacing their motorised transport (e.g., bus and car) with walking or cycling: For example, one participant wrote, *“Walking to local shops and parks when before I would have used the bus at least one of the ways.”* (71y Male, Rest of Scotland). It was also positive to note that some participants indicated that they had intended to sustain this particular change: *“I haven’t used public transport since lockdown. I have walked everywhere and I [intend] to continue to do so.”* (68y Female, Rest of Scotland).

Many participants also noted that they optimised or maximised their walking opportunities. This took many shapes; some participants reported replacing what would have been their work commute with walking, some reported deliberately taking longer routes to the shops or other destinations, and some reported how their walking pattern(s) had changed: *“do a nightly walk roughly equivalent in distance to my usual commute to work”* (60y Male, Rest of Scotland) and *“Each morning I walk to buy a newspaper during lockdown I have extended this walk. Instead of going directly to the shop my walk now takes about an hour each day instead of about 10 minutes.”* (62y Male, Rest of Scotland).

Participants reported that access to facilities (or the lack thereof) was a major reason for the change in their physical activity. It was also a reason for the introduction of new physical activities. Several participants did mention that they missed the gym along with the social interaction that often accompanied their exercise. However, it was positive to note that some participants were able to continue with their usual activities such as Pilates, dance, and aerobics classes which had moved to an online format. Additionally, some participants mentioned that where there was not an online alternative, walking or cycling served as a complementary exercise: *“I started doing daily exercise classes online instead of going to the gym. I’m doing a mix of Yoga, Pilates and Taichi. I’m also started to go for a walk as a routine.” (*64y Female, Rest of Scotland). However, there were also participants who reported that none of their usual activities were options, even online, so their modality of physical activity changed. For instance, *“Cycling or walking or both undertaken every day during lockdown with only two exceptions. Because other activity options were unavailable exercise opportunities became very important.” (*63y Female, no postcode provided), and *“I usually swim 5 or more days per week for at least an hour but with pool being closed I now walk instead”* (67y Female, accessible rural). Three other topics that came up far less frequently but are worth mentioning with reference to online physical activity specifically, included 1) internet connectivity issues; 2) lack of enthusiasm to start or attempt a new physical activity; and 3) lack of motivation to persist with a new physical activity.

Many participants reported health and injury as a reason for their change in physical activity. It is noteworthy that only two participants explicitly stated Covid19 when answering the questions about physical activity. These two participants reported that they had contracted Covid19 and had restricted their physical activity due to their recovery. One of the two participants mentioned that they had been hospitalized. Commonly mentioned injuries and health issues that affected physical activity included cardiac issues and musculoskeletal injuries, some of which occurred during the period of social distancing and were further affected by the restrictions. Examples include, *“I started walking more, but I have hurt my knee, so walking has been curtailed”* (67y Female, Rest of Scotland) and:

*“A few weeks before social distancing I had a heart attack and 2 stents fitted. Lock down precluded cardiac rehab classes. Cardiac physio sent exercise DVDs from cardiac rehab and [month] ago I was referred to local council. … I currently use the online videos”* (66y Female, Rest of Scotland).

It is also worth noting that several participants reported that social distancing had assisted with their injury management, and so although they were injured and/or recovering, the regulations on social distancing and lockdown afforded them time to recover and improve their physical activity: *“Lots more walking. Up to 6 miles a day. Weather dependent though. Had hip replacement in December and this had been FANTASTIC for it. And for me in general. Loving the activity.”* (66y Female, accessible rural).

The influence of shielding (or stay-at-home orders) on physical activity changes was mentioned by both participants who were shielding (due to their own health) as well as those who were residing with someone who was shielding (e.g., a child or partner); the role of caring is detailed later. It is important to note that responses regarding shielding did appear to be different depending on when the survey was completed (see Fig. 1 for shielding guidance and the restrictions physical activity in relation to timing of data collection). An additional point that was often mentioned alongside shielding was the type of housing or general space available for physical activity, which unsurprisingly was an important factor for individuals who were advised to not leave home for their safety. Participants wrote: *“Since I am shielded my only exercise is housework” (*88y Male, rest of Scotland), *“Shielding required a complete change in exercise behaviour. 12 weeks confined to house & garden so indoor cycling and brisk walks round garden. Now allowed out”* (67y Female, accessible rural), and *“Whilst shielding I started walking round garden as I couldn’t go out”* (64y Female, no postcode provided).

Several participants mentioned that their pets played a role in their change of or new physical activities during lockdown. One participant mentioned engaging in daily horse-riding exercise although dogs were the most commonly reported pet influence. For most participants that mentioned their dogs, a common theme was that they were either walking for longer and/or walking more frequently than before social distancing: *“I now walk my dog twice a day 7 days a week, instead of only 2 walks a day at weekends. I now walk approx 21 hours per week compared to 4 hours prior to lockdown”* (60y Female, accessible rural).

The first lockdown in Scotland occurred during the spring/summer months. Based on the responses of several participants, fair weather made outdoor physical activities such as walking and gardening possible. Conversely, ‘bad’ weather appeared to be a deterrent to being outdoors – particularly as the end of summer approached:

*“I haven't started any new physical activities. I walk almost every day round a park, to shops etc. I have been cycling a bit more recently. Part of the reason for that is the nice weather we have experienced in recent weeks. … My routine has not changed a lot because I am retired, and I walk as much as I can, anyway.”* (61y Female, accessible rural).

It was interesting to note how few responses included the element of time (or lack thereof) with regard to changes in physical activity relative to other responses detailed above. Based on the volume of responses, it appeared that in the context of all pandemic-related challenges, time was not a major barrier for adults aged 60 years and over. However, when time was mentioned, it was both a facilitator (where someone reported having more time) and a barrier (where someone reported having less or no time), albeit less frequently. In several instances, time was linked to employment (or lack thereof due to being furloughed) or other responsibilities: *“Knowing that I was going to be home all day for a rather long time, I decided to incorporate a workout into my daily schedule.”* (65y Female, Rest of Scotland).

Beyond the reported changes in physical activity due to social distancing, we were interested in responses that described successful strategies to maintain physical activity as well as responses that reflected positive changes related to physical activity that people made during social distancing (and/or because of stay-at-home guidance and closure of leisure facilities). These anecdotes ranged from participants’ positive experiences in discovering something new, to enjoying domestic but physical tasks, to seeing measurable improvement in their physical ability, and in several instances, participants reported setting goals or targets and reported how this motivated them: For example, *“I have decided to walk at least 10k steps daily over lockdown and I have done so on the vast majority of days”* (72y Female, rest of Scotland), and:

*“I started exercising accompanied by better diet and using a treadmill. Going for more walks has increased my fitness, lowered my weight and increased my mobility. The increased mobility has meant that I can now manage steeper inclines, walk faster and for longer periods. The change is that I'm actually exercising, which I found almost impossible before. I didn't do anything physical because I was overweight and in discomfort. I've lost almost 1st 11 lbs and at almost 67 yrs old, I feel so much better. I probably wouldn't have done it if lockdown hadn't happened.”* (66y Female, accessible rural)

and

*“I have a gym membership but [don’t] like classes because they are too busy so online zoom has been brilliant for me. I can see a change in my shape for the better. I am going to continue running several times a week. I didn't run at all before lockdown”* (66y Female, rest of Scotland)

Regarding the location of physical activity, the most frequently identified location of physical activity for participants was the local area (*n*=646, 54%), followed by in the house (*n*=559, 47%), in the garden (*n*=512, 43%), and online (*n*=195, 16%). One hundred and sixty-nine participants (14%) selected the ‘other’ option. In these responses, the most frequently reported ‘other’ options included golf courses, blue space (including the beach and lochs), in the woods or hills, and places of work. Several participants used this space in the survey to elaborate on their physical activity location and the reasoning: *“I live in the country and have horses and sheep and dogs do plenty physical exercise!”* (73y Female, accessible rural), and “*At work. I'm employed in grounds maintenance. It can be very physical.”* (64y Male, rest of Scotland).In response to the final question of the survey, for which participants were prompted to add anything that they felt had not been covered in the survey, the overwhelming majority of responses included stories of frustration, grief and longing to see grandchildren (that have been described in detail elsewhere [25]). However, several participants did elaborate on their physical activity, often linking it to their social activity and other themes identified in the physical activity-specific questions, such as health and injury: “*I feel much less fit and strong, and am of an age where regaining those may prove difficult...*” – 81y Female, rest of Scotland) and:

*“I had covid and now have long tail covid. My family are wary that I may still be infectious. My physical activity is very curtailed. I used to row competitively, cycle, yoga, walk everywhere. I can only manage slow, short walks. I am signed off sick from work.”* (62y Female, rest of Scotland).

Several participants also elaborated further on their lived experience and how the time of social distancing paved the way for a positive future, sometimes through their strategies to remain physically active:

*“I had time on my hands during lockdown and was worried about my weight and my diabetes putting me at greater risk of a bad outcome if I contracted covid-19. I decided to join an online health and fitness plan, which has been a great success. In 2 months I have lost over 2 [stone]. I now walk every day routinely more than 10,000 steps a day. I have also joined the One Million Step Challenge. In addition to this, I have discovered another strand of support in my online group and online coach. As a result of joining this programme, I am now [eating] only wholefoods and following a low-carb diet. I believe my blood sugars are back under control, I no longer have sugar cravings, I am sleeping much better and I am now not nearly as anxious as I was previously.”* (61y Female, rest of Scotland),

## Discussion

This study reported on the volume, perceived changes of, and associated factors with physical activity of Scottish older adults during the period of stay-at-home orders and social distancing guidelines between May and July 2020. The findings from this study add to evidence describing how people cope and adjust their physical activity and other associated behaviours in periods where the risk of reduced and/or restricted physical activity is high. Although specific to the Covid19 pandemic, this study has wider implications of helping understand the impact of social distancing on older people’s physical activity. The key findings include: 1) on average, participants reported high levels of walking and MVPA, but also very low levels of strength training, 2) the majority of participants experienced changes to their usual (pre-social distancing) physical activity but that reasons for changes in physical activity and how these came about (changes in volume, frequency, modality, intensity, or a combination of these) were highly variable; and that 3) physical activity level, time spent walking, and sleep were found to be associated with health-related quality of life. These three key findings should be considered in recovery strategies targeting older adults following the pandemic.

Regarding the first key finding, this study reports that during social distancing, most of our participants reported physical activity levels indicative of meeting national physical activity guidelines [44]. However, this is inclusive of MVPA as well as walking. When considering MVPA exclusively, as in the Scottish Health Survey [23], 42% of our participants over 60 years were ‘highly active’, and therefore meeting the MVPA guidelines. This is comparable to the Scottish Health Survey data collected between August and September 2020, where 47% of adults between the ages of 45 and 64 years, 42% between 65 and 74 years, and 34% over 75 years met the physical activity guidelines. Further, only 27% of those advised to shield (via letter/text) reported meeting guidelines in August/September 2020 [23], indicating that as the pandemic persisted and social distancing guidelines continued, for certain older adults in Scotland, there appeared to be an overall negative impact on physical activity levels. It is interesting to note that a decline in physical activity due to social distancing was not perceived by around 60% of our participants, who indicated that their MVPA and walking had remained about the same/more than before social distancing. However, this may be because our sample was represented by a greater number of older adults between 60 and 69 years, half of which reported having one medical condition. It is therefore likely that we did not collect data from many who were shielding (i.e., 70+ years and/or with a particular medical condition). In comparison to other Scotland-based studies, one longitudinal study in adults focussed on changes of movement behaviours (physical activity, sleep, and sedentary behaviour) during the pandemic. The changes in physical activity (walking and MVPA) are somewhat consistent with our findings. First, regarding MVPA changes from pre- to mid-lockdown, one study reported ±30% of the participants reported a positive change for MVPA, 47% maintained MVPA (although 25% were at a high level), and 23.7% reported less MVPA engagement [45]. Secondly, walking from pre- to mid-lockdown increased in 24% of the participants, 49% maintained their walking levels (38% maintained a high level of walking) and walking decreased in 28% [45]. Some of the differences between our findings and those reported by Janssen and colleagues may be due to greater variation in the ages represented in the Janssen study; although importantly, their findings suggested that the changes in physical activity was dependant on type of activity. This too is reflected in our findings, particularly in the qualitative component where participants were able to provide more detail about how and why these changes occurred. This reinforces the need for tailored approaches when targeting physical activity in older adults, both during periods of isolation and post-pandemic [46].

Another important consideration is that engagement in strength training was very low. Only 12% of the older adults in our study reported engaging in strength training on two or more days a week. This was likely due to lack of access to typical strength training equipment in the home, and (likely to a greater extent) the closure of gym facilities. This was indicated in our qualitative findings. It is also worth noting that over 70% of participants indicated that their strength training levels were like pre- social distancing levels, and almost a quarter reported less engagement in strength training. This indicates that the low engagement in strength training is persistent, and restrictions caused further deterioration in strength training engagement. This level of strength training appears even lower than the already reported low levels in Scottish data pre-pandemic, for example, from the Scottish health survey where among over 65-year-olds, only 31% of men and 24% of women met the muscle strengthening guideline [47]. These reportedly low levels of strength training are a concern due to age-related declines in muscle and bone strength associated with a loss of functional capacity, mobility, quality of life and independence [48, 49]. Additionally, low levels of strength and balance ability increase the risk of physical frailty and falls in older adults [9, 50]. This is especially important in situations where older adults have been more sedentary (e.g., 60% of older adults in this study reported sitting more than before social distancing) and would experience the greatest benefit from increasing strength/engaging in strength training. These findings suggest that more work is needed to encourage strengthening activities generally among older adults, and particularly during times of restricted physical activity or where access to opportunities to engage in strength training is compromised [51].

On the positive side, it is promising that many participants had identified strategies to remain physically active. This accords with findings that adolescents or adults who employed digital resources were more likely to undertake exercise classes and meet PA guidelines [52]. This is important given associations between low levels of physical activity and worse wellbeing [53, 4]. Over 60% of participants reported engaging in at least similar levels or more of moderate intensity physical activity and walking. Further, close to three-quarters reported the same or greater levels of light intensity physical activity. Based on this study, the strategies used to do so included increasing either the volume and/or frequency of walking. This was through active travel, or with pets, or for the sake of being outdoors participants spent more time active outdoors in their garden. For some, although to a lesser extent, this was through being active online. These points are important to consider in terms of providing recommendations on how to maintain physical activity, especially in light of results from a recent systematic review reporting that MVPA may improve vaccine potency [53]. First, the most frequently reported new or changed physical activity was active travel, with some participants indicating that this change was one they intended to sustain beyond the pandemic, thus support to encourage active travel in all ages would be beneficial. There are already examples of this in major cities in Scotland and for both physical activity and environmental reasons such as pop-up cycle lanes and car free plans. Second, it was encouraging that there was some level of engagement in online exercise classes including aerobics, Pilates, Yoga and TaiChi. However, this was applicable to less than 20% of our participants even though our sample had slightly greater representation of participants from low deprivation areas. Although further formative work is needed on the use and development of online materials for use in older adults, our findings suggest that future online exercise sessions and/or physical activity resources might not be a direct replacement for in-person physical activity but could complement existing face-to-face programmes. Issues relating the internet connectivity were noted in this study and whilst this was a largely affluent sample, further consideration of digital poverty would need to be addressed to make online physical activity opportunities equitable.

Thus, this study emphasises the value in exploring the role that digital technology may have in older adults’ physical activity. Based on the present findings, the potential options are varied. For some, switching their physical activity to online activities helped them to maintain their physical activity levels. Thus, educating older adults to increase their digital literacy level could be valuable for physical activity [54]. Increased screen time reporting suggests that the use of technology by over-60’s may have increased, so the use of online classes targeting older more sedentary populations may be better received post-pandemic. For older adults who are more digitally ‘literate’, online-based physical activity may be an effective complementary tool for increasing current physical activity or introducing new kinds of physical activity; it may also serve as a motivational tool. Older adults who are not partial to or skilled with screen-based physical activity may rather benefit from using their screens to socialise and could be encouraged to incorporate walking into their screen-based social activity, although educating older adults to increase their digital literacy level to increase physical and social activity could still be really important [55]. It would also be valuable to examine the potential for these types of interventions within specific sub-groups such as people with dementia or carers, as well as seeking to understand the mechanisms underlying the link between physical activity and wellbeing better in these groups.

### Strengths and limitations

This study used a large sample of Scottish adults to explore perceived changes in physical activity due to social distancing guidance in response to a pandemic. However, there are also several limitations that should be considered. First, the nature of the study design did not allow for the exploration of change over time and so the results presented are cross-sectional and perceived changes obtained through self-report, it was not possible to explore objective change, potential mediation of effects, or causality. However, this study design was appropriate because the aim was to provide rapid evidence surrounding the impact of social distancing, rather than how this altered over time and a second questionnaire was thought to be too burdensome for older adults during this time. Additionally, the use of devices such as accelerometers to measure physical activity, sleep and sitting time was not feasible. Second, there were a number of limitations within the survey that are worth noting for future studies: 1) the known limitations with self-report measures of physical activity such as the IPAQ; 2) ambiguity in the two open-ended PA questions (change in physical activity versus new physical activity), which often meant that participants provided two answers that were somewhat incomplete if not considered simultaneously (e.g., a participant might have said “no” to doing new PA but then provided a lengthy answer regarding the change in PA). Similarly, participants sometimes gave conflicting information (e.g., saying “no” to changed activity but saying that online PA was “new” and a change from in-person to online); 3) as mentioned, the questions pertaining to screen time did not specify whether the screen time was sedentary or active, or whether the screen was being engaged independently (e.g., engaging with a mobile device in a social manner may differ from have a television on in the background while cooking); 4) further detail about duration, volume, or intensity of strength training would have provided greater insight into resistance exercise. Additionally, participants were not asked about balance activities. Further, given the survey design, the qualitative data were not as rich as would be achieved from focus groups or interviews. However, we were still able to gather considerable useful explanatory information to complement our quantitative data, and the design suited the research restrictions due to Covid19. Finally, our change questions specifically related to social distancing which was only one of the components of government restrictions, so it could be argued that while our phrasing was about social distancing, our data might reflect the effects of the other restrictions at the time. However, we would argue that the government’s advice to socially distance was restrictive and had an impact on both social and physical activity in this age group. Exercise and social centres were closed due to not being able to maintain distancing, and shielding was an extension of social distancing for those most at risk. Consequently, it was deemed best to focus on social distancing and define in the survey what this meant in terms of restrictions to meeting in groups, face to face gatherings, and staying at home.

## Conclusion

This study has highlighted a range of positive and negative effects on participants’ physical activity and wellbeing as the result of the social distancing associated with Covid19. For many older adults in this study, being able to walk, whether around their gardens, to the shop or around the local area helped them cope with the restrictions of social distancing and keep active. Physical activity and walking were found to be positively associated with wellbeing, supporting that the promotion of access to green space, particularly in urban settings, could have an important impact on wellbeing. This study also emphasises the importance of promoting the value of strength training in older adults, particularly when access to gyms is limited. These findings are important because they provide lessons on how to better promote health and wellbeing for older people in the future. Specifically, there are lessons regarding how best we can help older people use existing resources and develop new ones to promote health and wellbeing in the future, both within and out with pandemic situations.

## Supplementary Information

**Additional file 1.**

**Additional file 2.**

## Data Availability

The datasets during and/or analysed during the current study available from the corresponding author on reasonable request.

## Abbreviations

IPAQ: International physical activity questionnaire
MVPA: Moderate- to vigorous-intensity physical activity
PA: Physical activity
