# Supplementary figures and images for: “… Exercise opportunities became very important”: Scottish older adults’ changes in physical activity during Covid19’
Source: Eur Rev Aging Phys Act. 2022 Jul 2;19:16. doi: 10.1186/s11556-022-00295-z (PMC9250220; doi:10.1186/s11556-022-00295-z)

**S2: Supplementary spline plots** depicting associations with loneliness (UCLA) and wellbeing (EQ5D):


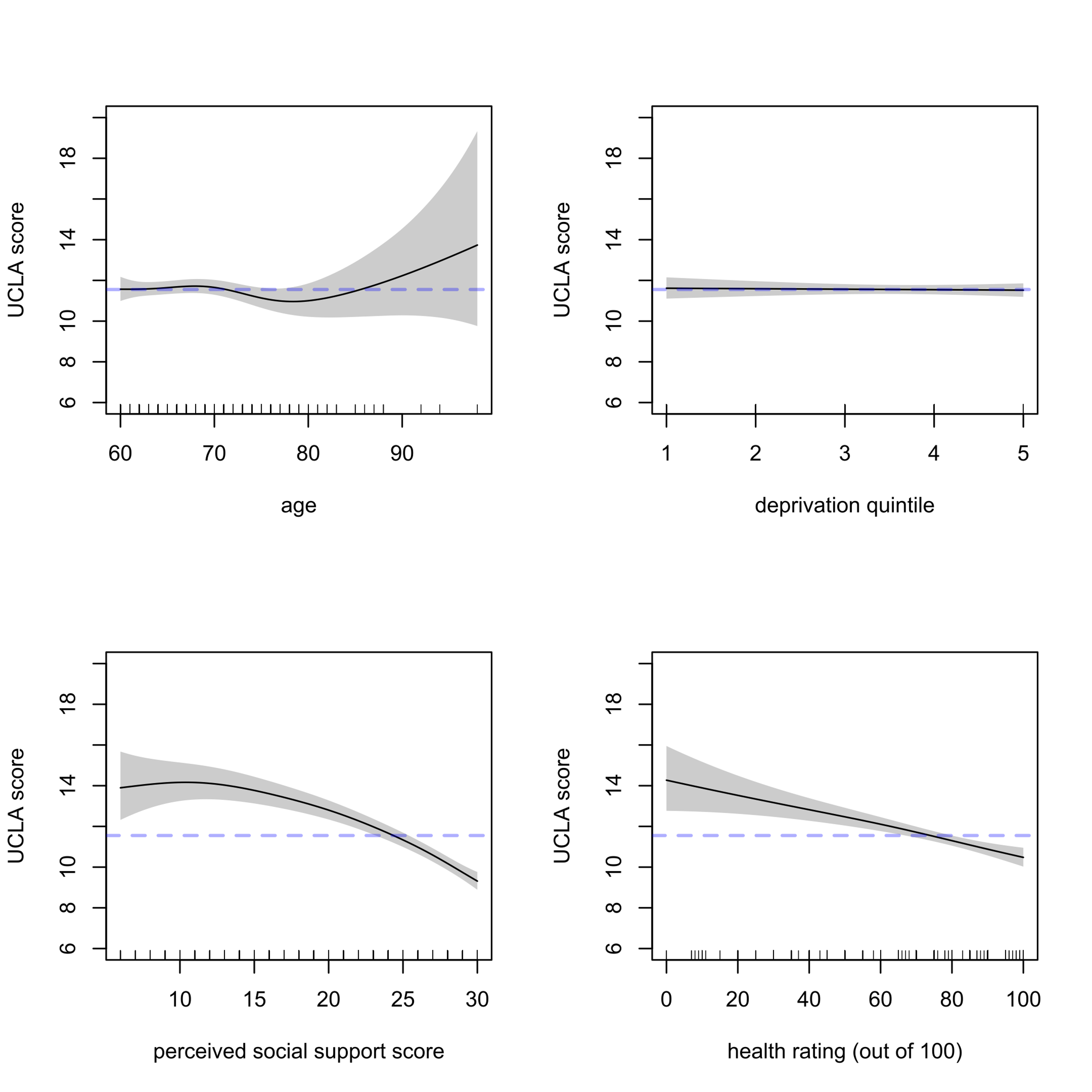


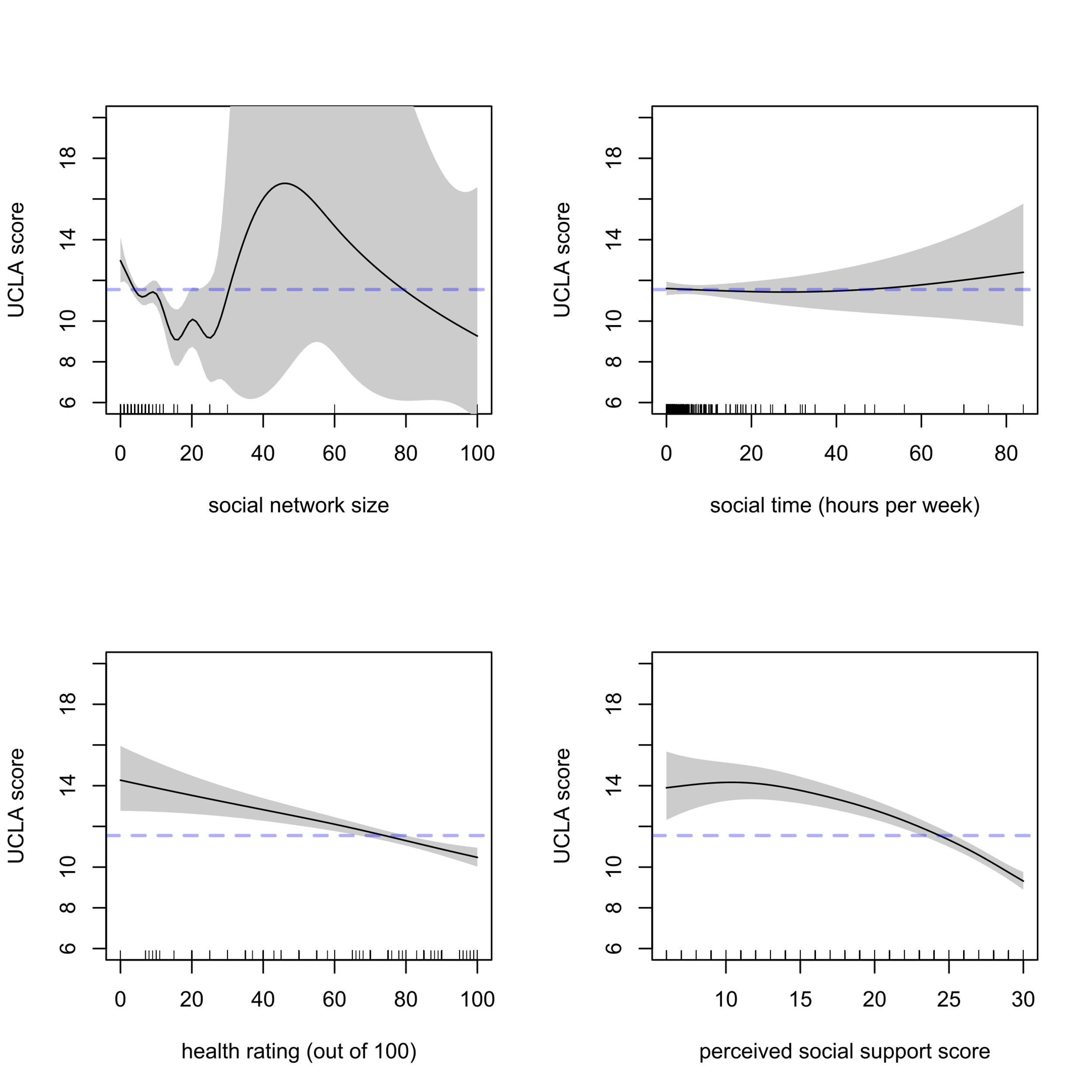


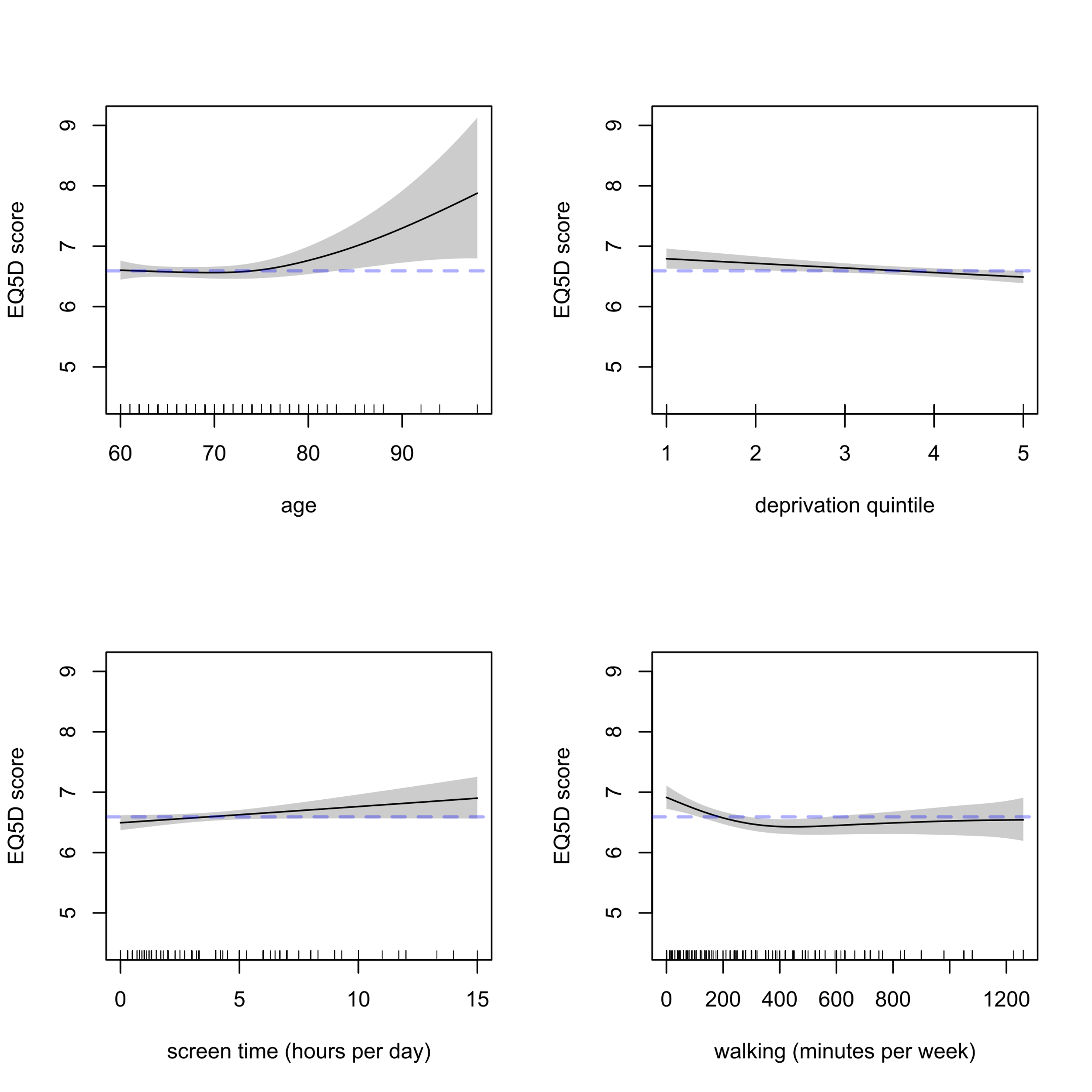


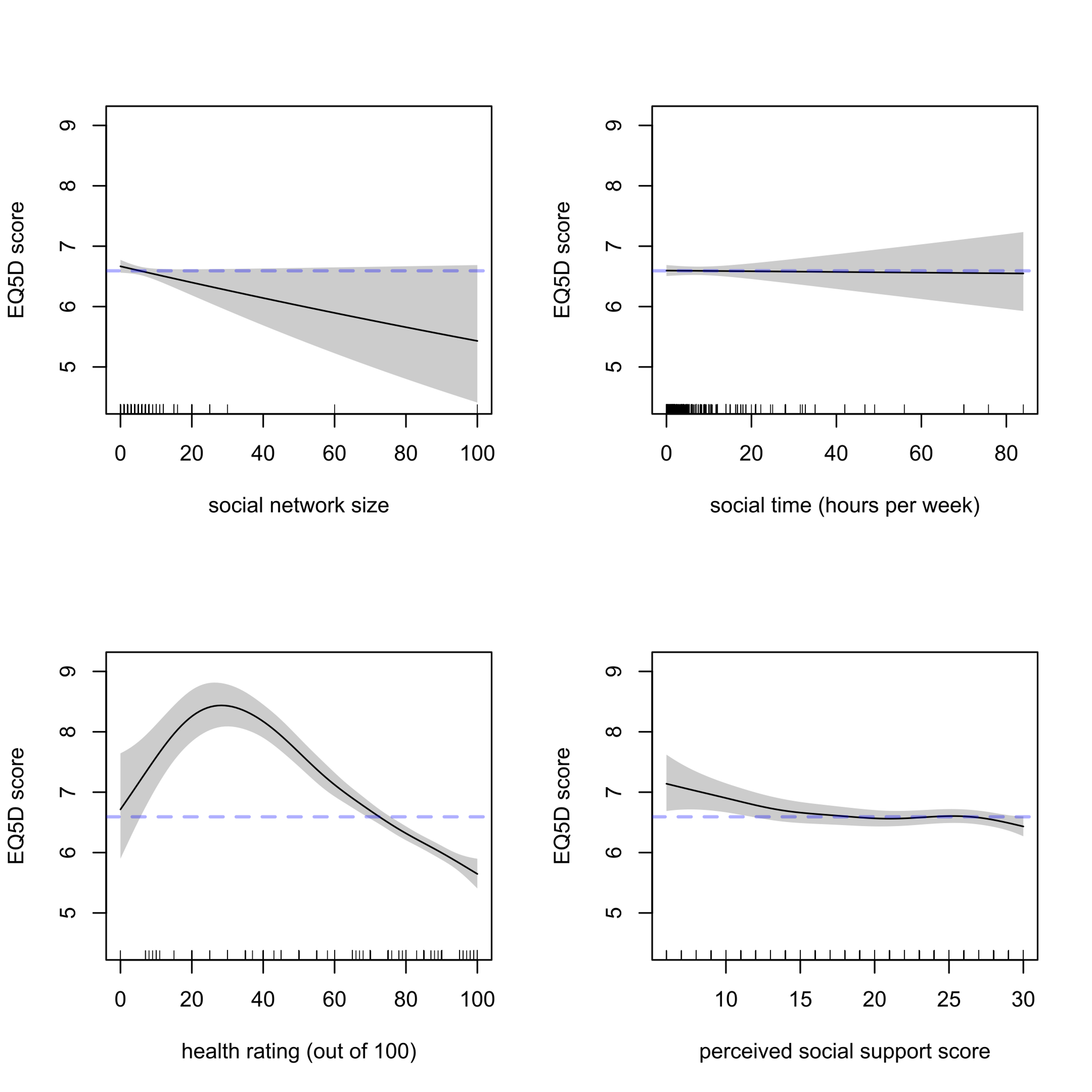

Supplement: Supplementary file 2 — Additional file 2. [file 11556_2022_295_MOESM2_ESM.docx]
